# Effects of bromelain on the quality of smoked salted duck

**DOI:** 10.1002/fsn3.2422

**Published:** 2021-06-24

**Authors:** Ziqing Ye, Jian Zhang, José M. Lorenzo, Mutian Zhang, Wangang Zhang

**Affiliations:** ^1^ Key Lab of Meat Processing and Quality Control Ministry of Education Jiangsu Collaborative Innovation Center of Meat Processing and Quality Control College of Food Science and Technology Nanjing Agricultural University Nanjing China; ^2^ Centro Tecnológico de la Carne de Galicia Ourense Spain; ^3^ Área de Tecnología de los Alimentos Facultad de Ciencias de Ourense Universidad de Vigo Ourense Spain; ^4^ Nanjing Cherry Duck Industry Company Nanjing China

**Keywords:** bromelain, flavor, quality, smoked salted duck

## Abstract

This study was aimed to assess the effects of bromelain on the eating quality of smoked salted duck. Whole ducks were marinated with different doses of bromelain (300 U/g, 600 U/g, 900 U/g, 1,200 U/g and 1,500 U/g), while the group without bromelain was considered as control (CK). After the production of smoked salted duck was completed, the pH, color, texture, electronic tongue detection, thiobarbituric acid reactive substances (TBARS), sodium dodecyl sulfate‐polyacrylamide gel electrophoresis (SDS‐PAGE), and mass spectrometry analysis were determined. The results showed that, compared to CK, the pH, TBARS and hardness values in 900, 1,200 and 1,500 U/g groups were reduced. The cohesiveness and the springiness were increased while the values of b* were decreased in all bromelain treatments (*p* < .05). The SDS‐PAGE and mass spectrometry analysis indicated that myosin and actin were further hydrolyzed into small‐molecule proteins by bromelain. Electronic tongue detection showed that the umami, the saltiness and the richness of smoked salted duck were enhanced, while the bitterness was reduced at the dose of 900 U/g. Thus, bromelain improved the eating quality of smoked salted duck in particular at the level of 900 U/g.

## Graphical abstract

This study was aimed to assess the effects of bromelain on the eating quality of smoked salted duck. The addition of bromelain improved the eating quality of smoked salted duck, in particular, at the level of 900 U/g.

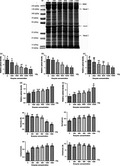

## 1 INTRODUCTION

Nowadays, many cured meat products are produced from different raw materials and under different processing conditions (Lorenzo et al., 2008; Toldra, 2006). During the processing of cured meat products, chemical and biochemical changes lead to produce a large number of volatile compounds which contribute to their characteristic flavor (Shi et al., 2021). Smoked salted duck is one of the Chinese traditional cured meat products which are largely favored by consumers in China due to its unique flavor (Li et al., 2011). Traditionally, the smoked salted duck is produced by marination and slowly air‐dried process and then is matured under natural conditions at 4–10℃ for approximately one month (Luo et al., 2008). Therefore, as to shorten the production cycle, high temperature (45–60℃) is generally used instead of natural air drying in modern factories (Zhang et al., 2013). However, the efficiency of endogenous enzymes in duck meat is easily inhibited at high temperature (Perez‐Santaescolastica et al., 2018), which reduces the degree of protein hydrolysis, and thus induces flavor deficiency and tough texture (Feng et al., 2014). In addition, the lipid and protein oxidation in dried cured meat products would be accelerated at high temperatures leading to the deteriorated flavor (Dominguez et al., 2019; Rivas‐Canedo et al., 2021). Thus, softening texture and enhancing flavor are used to improve the eating qualities of smoked salted duck for industry (Lorenzo et al., 2013).

Proteolysis is considered as an important biological and chemical change during the manufacture and ripening period of cured meat products resulting in improved texture and flavor (Chen et al., 2021; Zhang et al., 2017). Because the enzyme activity from microorganisms is relatively low, the proteolysis is mainly catalyzed by endogenous enzymes (Keska et al., 2017). Endogenous enzymes hydrolyze proteins in ducks to generate peptides and free fatty acids (FFAs), which are considered as the important flavor precursors (Huang et al., 2014). However, endogenous enzymes could be inhibited by high drying temperature (Zhang et al., 2017). Therefore, the treatment of exogenous enzymes is particularly important in the modern process, which can promote protein hydrolysis and form flavor precursors. Setiadi et al. (2019) added the transglutaminase enzyme to duck meat showing that it significantly improved the texture profile parameters (hardness, springiness and cohesiveness) and organoleptic parameters (taste, aroma and color). Feng et al., (2018) used bromelain to tenderize golden pomfrets (*Trachinotus blochii*) and found the concentrations of FAAs were significantly increased, especially glycine, alanine, lysine, and methionine. Flavourzyme was used to produce Cantonese bacon, and the flavor compounds were increased (Zhang et al., 2017).

Bromelain can be extracted from fruits and stems of Bromeliaceae, mainly from *Ananas comosus* (Ramli et al., 2018). Bromelain is a plant protease in the sub‐group of thiol (cysteine) proteinases such as papain from papaya and ficin from figs. As a conveniently available material, bromelain has attracted more and more attention in the fields of medicine, biotechnology, and food due to its exploitable characteristics (Campos et al., 2020). Bromelain has been reported to have excellent activity in improving the tenderness and enhancing the flavor of fresh meat (Chaurasiya et al., 2015; Sonklin et al., 2018; Xu et al., 2020). Feng et al., (2017) have reported that bromelain could accelerate the proteolysis of golden pomfret protein, softening texture and enhancing flavor of fish balls. Zhao et al., (2020) found that bromelain‐treated beef had higher level of free amino acids and ketones. However, few information is available regarding the effects of bromelain in smoked salted duck. Thus, the purpose of this study was to determine the quality changes of smoked salted duck with bromelain‐assisted marination.

## 2 MATERIALS AND METHODS

### 2.1 Preparation of smoked salted‐duck meat

The frozen ducks (Cherry Valley ducks) were bought from Henan Huaying Agricultural Development Company Limited (Henan, China). For thawing, the 36 samples were kept at 4℃ for 24 hr and randomly allocated to six groups (six whole ducks per group) before use. The bromelain‐treated groups were separately submerged in 24 L different concentrations of bromelain solutions (300 U/g, 600 U/g, 900 U/g, 1,200 U/g and 1,500 U/g; Jiangsu Xinrui Biological Technology Company) for marination (TW20, JULABO) at 50℃ for 2.5 hr (Chen et al., 2016). After incubation with the salt solution (10%) at 4℃ for 24 hr, samples were baked in a constant temperature and humidity incubator box for 17.5 hr (40℃, relative humidity (RH) 80% for 2 hr; 70℃, RH 40% for 30 min; 40℃, RH 60% for 15 hr; KBF115pgm; Binder), smoked for 40 min (65℃, RH 55%; Ti3000; Fessmann), and air‐dried for 72 hr (8℃, RH 50%; KBF115pgm; Binder) (Wang et al., 2009). Afterwards, the samples were stored at −80℃ in a freezer (DW‐86L626; Haier) until further analysis.

### 2.2 Physiochemical indexes

The color of smoked salted‐duck breasts was measured using a chromameter (CR‐400; Minolta Camera) under illuminate C, 2° standard observer and 8 mm diameter of aperture (Zhao et al., 2018). A standardized white tile plate (L* (lightness) = 96.86, a* (redness) = − 0.15, b* (yellowness) = 1.87) was used to calibrate the chromameter before the measurement and then the model was adjusted to the L*, a* and b* system.

The pH values were determined with a pH meter (Hanna HI9025c, Hanna Instruments, Amorim, Portugal). Briefly, 2 g of duck samples (duck breast:duck leg = 1:1) were homogenized (5,000 rpm, 3 × 20 s; PD500‐TP; Prima) with 15 ml of distilled water (MUL‐9000XILIE; Millipor) at 20℃ and then kept still for 15 min before measurement (Gokoglu et al., 2017).

Cooking loss was measured by weighing each duck breast (20 g ± 0.1 g) sample before and after cooking. Cooking loss (%) = ((weight of raw meat‐weight of cooked meat)/weight of raw meat) × 100.

The measurement of moisture content was performed according to the method of Shi et al., (2020). Two grams of duck samples composed of duck breast and leg (1:1) were put into a 25 ml beaker which was previously dried. The beakers with 2 g of duck meat were kept at 105℃ for 12 hr. Moisture content (%) = ((original weight‐weight after being dried)/original weight) × 100.

### 2.3 SDS‐PAGE

Myofibrils of duck samples were extracted at 4℃ following the method of Chou et al., (1996) with some modifications. The 0.5 g of duck samples were weighed and homogenized (8,000 rpm, 3 × 30 s; PD500‐TP; Prima) with 5 ml of the standard solution buffer (100 mM phosphate buffer, pH 7.0, 2% SDS). After centrifugation at 4℃ (7,500 g, 10 min; Avanti J‐26S XP; Beckman Coulter), the concentration of the protein in the supernatant was detected with a BCA kit (Thermo, Pierce). Then, the protein concentration was adjusted to 10 μg/μl with the standard solution buffer. The same volume of diluted sample liquid with the loading buffer (10 mM Tris‐HCl, 10% glycerol, 2.5% SDS, 1% β‐mercaptoethanol, and 0.01% bromophenol blue) was mixed (30 s; VM‐03RU, Crystal) at 25℃. Then, the mixed liquid was heated at 95℃ for 5 min (TW20, JULABO).

The 10% polyacrylamide gels (Bio‐Rad Laboratories) were used, and the 50 μg protein samples were loaded in each lane. The samples were run with a SE 400 slab gel electrophoresis equipment (Bio‐Rad Laboratories). Gels were operated at the voltage of 80 V for 30 min and then 120 V for 60 min. Molecular weight markers were used as protein standards ranging from 10 kDa to 250 kDa (ThermoFisher). The band intensities were determined by the Quantity One software (Bio‐Rad Laboratories).

### 2.4 NanoLC‐ESI‐MS/MS

Referring to the method of Yu et al., (2016), NanoLC‐ESI‐MS/MS equipment was used to separate and identify protein composition. The high‐pressure liquid chromatography (HPLC) system (1,100, Agilent) with a 75 um and 8 cm in length was used with house‐packed reverse‐phase C18 capillary column (ThermoFisher). The particle size of the C18 was 3 μm, the pore size was 300 Ä, and the time of sample injection was 20 min. The HPLC solvent I was 97.5% ultrapure water (MUL‐9000XILIE, Millipor), 2% acetonitrile and 0.5% formic acid. The HPLC solvent II was 90% acetonitrile, 9.5% ultrapure water, and 0.5% formic acid. The gradation time for solvent II was 60 min from 2% to 90%. The time of sample loading and the time of column washing were 20 min separately. The typical sample injection volume was 3 μl. After separation, the column flow rate was approximately 800 nl/min.

The HPLC system was connected in series with electrospray ionization (ESI), and the samples were eluted by HPLC directly into the mass spectrometer and then ionized by ESI method. The capillary temperature was 100℃, and the ionization voltage range was 1.5 kV–1.8 kV. The data‐correlation mode was set as the mass spectrometer mode. The MS/MS data were obtained through the dissociation induced by low energy collision. The mass range of microscan was 350 am to 1,650 am with 33% default collision energy. The dynamic exclusion function was set to a repeat count 1, the exclusion width was 4 Da, and the exclusion duration was 1 min. The ProtQuest software package from ProtTech was employed to search the UniProt protein database using mass spectrometry data.

### 2.5 Texture profile analysis (TPA)

The TPA could obtain the texture characteristic parameters related to human sensory evaluation. Before performing the analysis, ducks’ breasts were packaged in vacuum bags, heated at 80℃ for 40 min (TW20, JULABO, Germany), and then carved to hexahedron (1 cm × 1 cm × 1 cm). The TPA was performed according to the method of Feng et al., (2017) with slight modifications. Hardness, springiness, cohesiveness, and chewiness of samples were determined at 25℃ with a cylindrical probe (P/50) of the texture analyzer (TA‐XT2i, Stable Micro System). The conditions were as follows: (1) 50% strain; (2) both pretest speed and test speed were adjusted to 2.0 mm/s; (3) post‐test speed was adjusted to 5.0 mm/s. It is worthy of noticing that two presses against each sample could result in two peaks. The parameters that were measured for TPA were the following: hardness (Hd) = peak force (N) required for first compression; chewiness (Cw) = Hd × Ch × Sp (N × mm); cohesiveness (Ch) = ratio of active work done under the second compression curve to that done under the first compression curve (dimensionless); springiness (Sp) = distance (mm) the sample recovers after the first compression.

### 2.6 Lipid oxidation

The analysis of TBARS was performed according to the method of Zhang et al., (2013). The 5 g samples were homogenized (10,000 rpm, 45 s; PD500‐TP, Prima, UK) at 4℃ with 25 ml of 7.5 g/kg trichloroacetic acid and 0.1 g/kg ethylene diamine tetraacetic acid. Then the mixture was centrifuged at 4℃ (12,000 g, 5 min; Avanti J‐26S XP, Beckman Coulter). This supernatant liquid (2 ml) was mixed (30 s; VM‐03RU, Crystal, China) with thiobarbituric acid (TBA, 20 mmol/L, 2 ml) at 25℃ and then heated in the water bath (TW20, JULABO) at 95℃ for 30 min. After the liquid was cooled by flowing water until reaching room temperature (25℃), the absorbance values were measured (Spectral Max M2e) at 532 nm. Based on the standard curve of 1, 1, 3, 3‐tetraethoxypropane, the results were presented as mg of malondialdehyde (MDA) per kg of meat sample.

### 2.7 Electronic tongue

To extract taste substances, approximately 25 g of minced (HM100, Grlnder, China) meat samples (duck breast: duck leg = 1:1) were mixed with 100 ml ultrapure water (VM‐03RU, Crystal). After being centrifuged (Avanti J‐26S XP, Beckman Coulter, USA), the mixture was filtered by filter paper (102, General Electric Biotech Hangzhou Company Limited) at 25℃. The aqueous phase was measured as electronic tongue samples (Zhang et al., 2019), and the Taste Sensing System (SA402B, Insent, Japan) was set. The sensors indicated bitterness, umami, saltiness, richness, astringency, and aftertaste of bitterness (aftertaste‐B). The taste substances in the aqueous phase could cause electric potential changes, and they were transmitted to the computer through the sensor. The transformation for test information in the taste analysis application converted the output of the sensor into taste information. The conversion file was selected according to the sensor used in the experiment without calculation and unit. The file required for this conversion was “Foodstuff‐Evaluation.ece”. Obtained values represented the intensity of taste properties.

### 2.8 Statistical analysis

Six samples were used in each treatment, and each sample was considered as a replication. Analysis on each duck was performed in triplicate (except for SDS‐PAGE and NanoLC‐ESI‐MS/MS), and the average was obtained. One‐way analysis (ANOVA) of SAS 9.2 was used to analyze the data with the bromelain concentration being considered as a fixed factor. The differences among the data were compared by Duncan's multiple range test. The significant level was considered at *p* < .05. The results were shown as mean values ± standard error.

## 3 RESULTS AND DISCUSSION

### 3.1 Physiochemical analysis

There were no significant differences in moisture content between CK and all bromelain‐treatment groups (Table 1, *p* > .05). The color of meat products is dependent on many factors such as a dry/moist environment, oxygen access, and the endpoint temperature (Ortuno et al., 2021). The value of L* was reduced in the 1,500 U/g concentration group (Table 1, *p* < .05). Compared with control, all bromelain treatments significantly reduced the values of b*, and the values of a* were reduced in 900 and 1,200 U/g groups (Table 1, *p* < .05). Due to the changes in pH, the moisture distribution and the texture of the meat were changed, and the reflection or absorption of light was changed, resulting in a decrease in L* (Forrest & Briskey, 2006). The value of a* was affected by the dynamic balance of myoglobin oxidation and oxymyoglobin reduction. The bromelain might degrade partial oxidase and reductase, resulting in changes in the value of a* (Faustman et al., 2010). Fat and the oxidative polymerization of carbohydrates could significantly affect the b* of meat (Wang et al., 2019). The reduced values of b* might be due to the antioxidant properties of peptides and amino acids from the protein degradation by bromelain thus decreasing the oxidation of fat, protein, and carbohydrates (Borrajo et al., 2020; Wang et al., 2019).

**TABLE 1:** Changes in physiochemical indexes of smoked salted duck

Enzyme concentration	0 U/g	300 U/g	600 U/g	900 U/g	1,200 U/g	1,500 U/g
L*	39.85 ± 0.29^ab^	38.89 ± 0.09^bc^	41.07 ± 0.29^a^	39.83 ± 0.28^ab^	40.55 ± 0.34^a^	37.77 ± 0.97^c^
a*	4.43 ± 0.23^bc^	5.03 ± 0.31^ab^	3.93 ± 0.11^cd^	3.24 ± 0.13^d^	3.16 ± 0.11^d^	5.35 ± 0.57^a^
b*	5.41 ± 0.35^a^	4.24 ± 0.17^b^	4.21 ± 0.16^b^	4.14 ± 0.20^b^	3.81 ± 0.29^b^	3.74 ± 0.24^b^
pH	6.12 ± 0.03^a^	6.07 ± 0.02^ab^	6.06 ± 0.01^ab^	6.02 ± 0.01^b^	6.00 ± 0.00^b^	5.90 ± 0.01^c^
Cooking loss (%)	9.20 ± 0.22^a^	9.13 ± 0.07^a^	8.94 ± 0.48^a^	8.35 ± 0.32^a^	8.80 ± 0.06^a^	6.30 ± 0.24^b^
Moisture content (%)	58.74 ± 0.36^a^	58.91 ± 1.30^a^	59.10 ± 0.56^a^	59.06 ± 0.45^a^	59.16 ± 0.24^a^	59.54 ± 0.61^a^

Note

Different superscripts (a, b, c, d) within a line indicate significant differences (*p* < .05).

The pH is highly important in meat products because it influences physical, chemical, and eating quality capabilities such as tenderness, juiciness, and water holding capacity of meat products (Grajales‐Lagunes et al., 2012). The pH values significantly decreased in 900, 1,200, and 1,500 U/g bromelain–treated samples compared to CK (*p* < .05). The protein hydrolysis by bromelain might result in releasing amino acids by removing amino groups. It was reported that removing amino groups would cause a reduction in pH (Gadekar et al., 2014). In addition, the deamination of proteins by enzymolysis releasing hydrogen atoms might decrease the pH values (Leygonie et al., 2011). The study of Buyukyavuz (2013) also found that the addition of bromelain decreased the pH of duck breast meat. Indeed, the author reported that the pH values were 5.97 in 1.5% bromelain–treated group as compared to 6.16 in the control group.

The cooking loss of 1,500 U/g bromelain–treated sample was significantly different from other treatments (Table 1, *p* < .05). The decreased cooking loss indicated that hydrophilic properties of protein in duck meat were improved by bromelain. These changes might result from the increased number of water binding sites being exposed increasing interaction between protein and water (Xiong, 2005; Zhang et al., 2017). Other study by Pietrasik and Shand (2010) found that the cooking loss of beef was decreased with the treatment by purified papain. Chaurasiya et al., (2015) also found that the 162.0 U/g purified bromelain reduced the cooking loss of beef from 49.7% (the control) to 47.2%.

### 3.2 SDS‐PAGE

**FIGURE 1:**
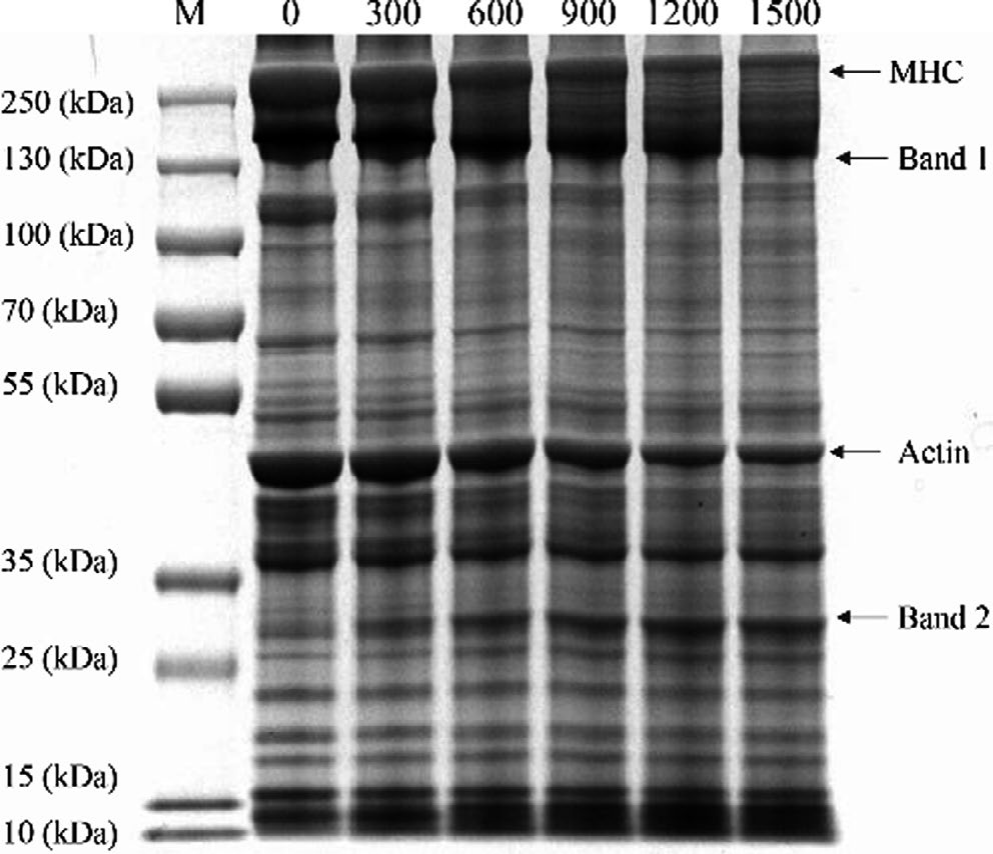
Marker (M), CK (0), 300 U/g group (300), 600 U/g group (600), 900 U/g group (900), 1,200 U/g group (1,200), and 1,500 U/g group (1,500)

**FIGURE 2:**
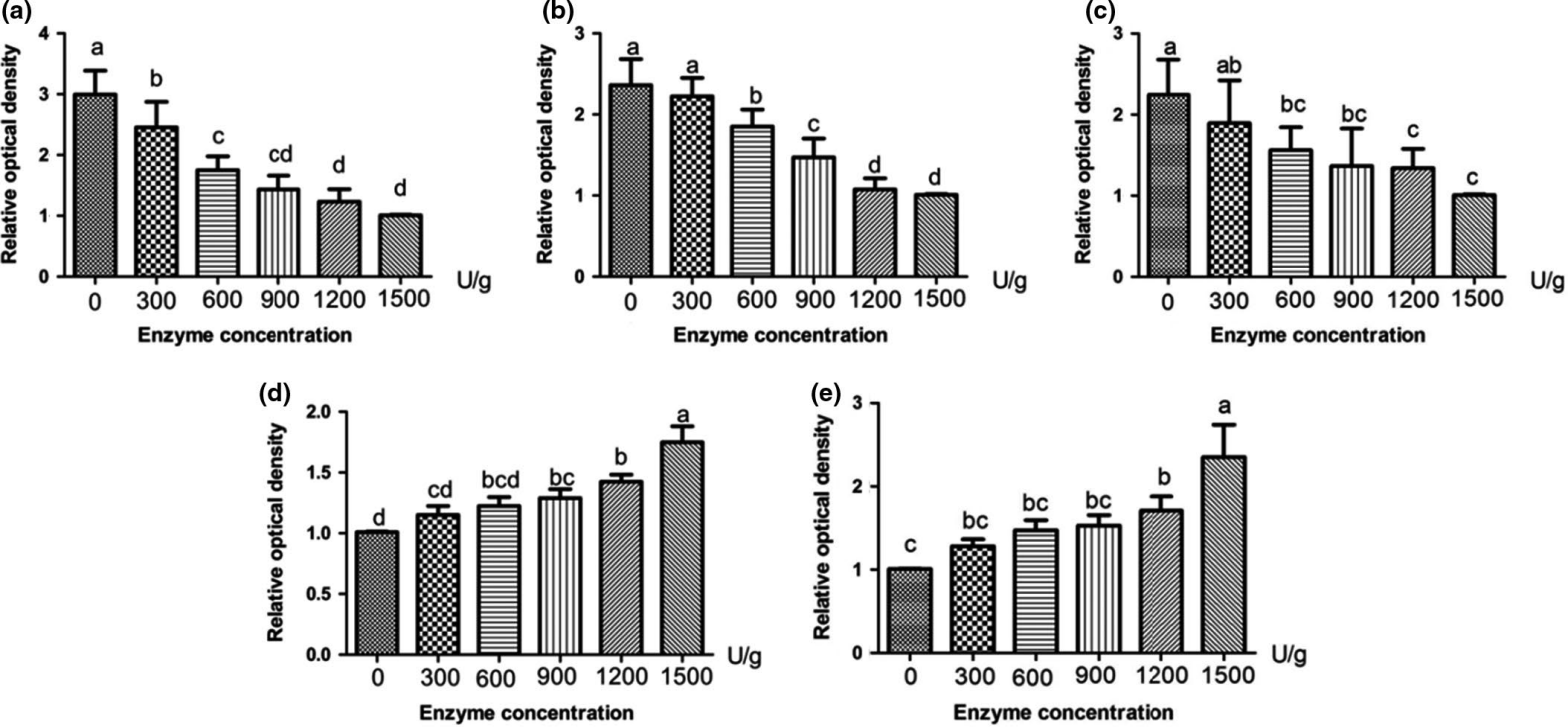
Changes in 250 kDa (a), band 1 (b), actinin (c), band 2 (d), and 10–15 kDa (e) of myofibrils of smoked salted duck. Different superscripts (a, b, c, d) indicate significant differences (*p* < .05). Relative optical density of 1,500 U/g and 0 U/g is the standard

The effects of bromelain on the protein changes of smoked salted duck could be seen from Figure 1 and Figure 2. The myosin heavy chain (MHC) and the actin were the major myofibrillar proteins in smoked salted duck. As the bromelain concentrations were increased, the reduction in the intensities of both MHC and actin were found. The intensity of 250 kDa MHC became significantly weak when the bromelain concentration was increased from 300 U/g to 1,500 U/g (Figure 2 A, *p* < .05). After NanoLC‐ESI‐M/MS analysis, the protein at band 1 was found to contain the type 2 myomesin and the type 6 myosin showed significant differences between CK and 600, 900, 1,200, and 1,500 U/g groups (Figure 2 B, *p* < .05). Actin was degraded significantly when the concentrations of bromelain were 600 U/g and above (Figure 2 C, *p* < .05). The protein of band 2 contained α‐1‐actin and β‐actin, which were significantly increased in 900, 1,200, and 1,500 U/g groups compared to CK (Figure 2 D, *p* < .05). The 10–15 kDa products were increased significantly in 1,200 and 1,500 U/g groups compared with CK (Figure 2 E, *p* < .05).

Bromelain could degrade the myofibrillar proteins leading to the myofibril fragmentation which changed physicochemical and structural properties of duck meat (Feng et al., 2018). In addition, the protein denaturation could occur at the same time during the drying process (Lorenzo et al., 2013). This was reflected in the reduced content of myosin and actin and the increased content of small molecular proteins, peptides, and FAAs. Feng et al., (2014) performed SDS‐PAGE on salt‐soluble protein of Chinese sausage treated with Flavourzyme. They found that the protein bands of MHC disappeared as the amount of Flavourzyme increased to 8 LAPU, 12 LAPU, 16 LAPU, and 20 LAPU, while the density of protein bands between 60 and 100 kDa increased. Similar protein degradation was also found in other study for duck breast muscle which was treated with ginger extract from fresh ginger rhizome (Tsai et al., 2012). During ginger extract marination, the amounts of MHC in 7‐day samples were reduced to approximate 84% of 0‐day samples, while the MHC amounts of the control were changed less than 10%. Xu et al., (2020) treated jumbo squid meat with bromelain and papain and found that more small peptides and short fragments were produced.

### 3.3 TPA

**FIGURE 3:**
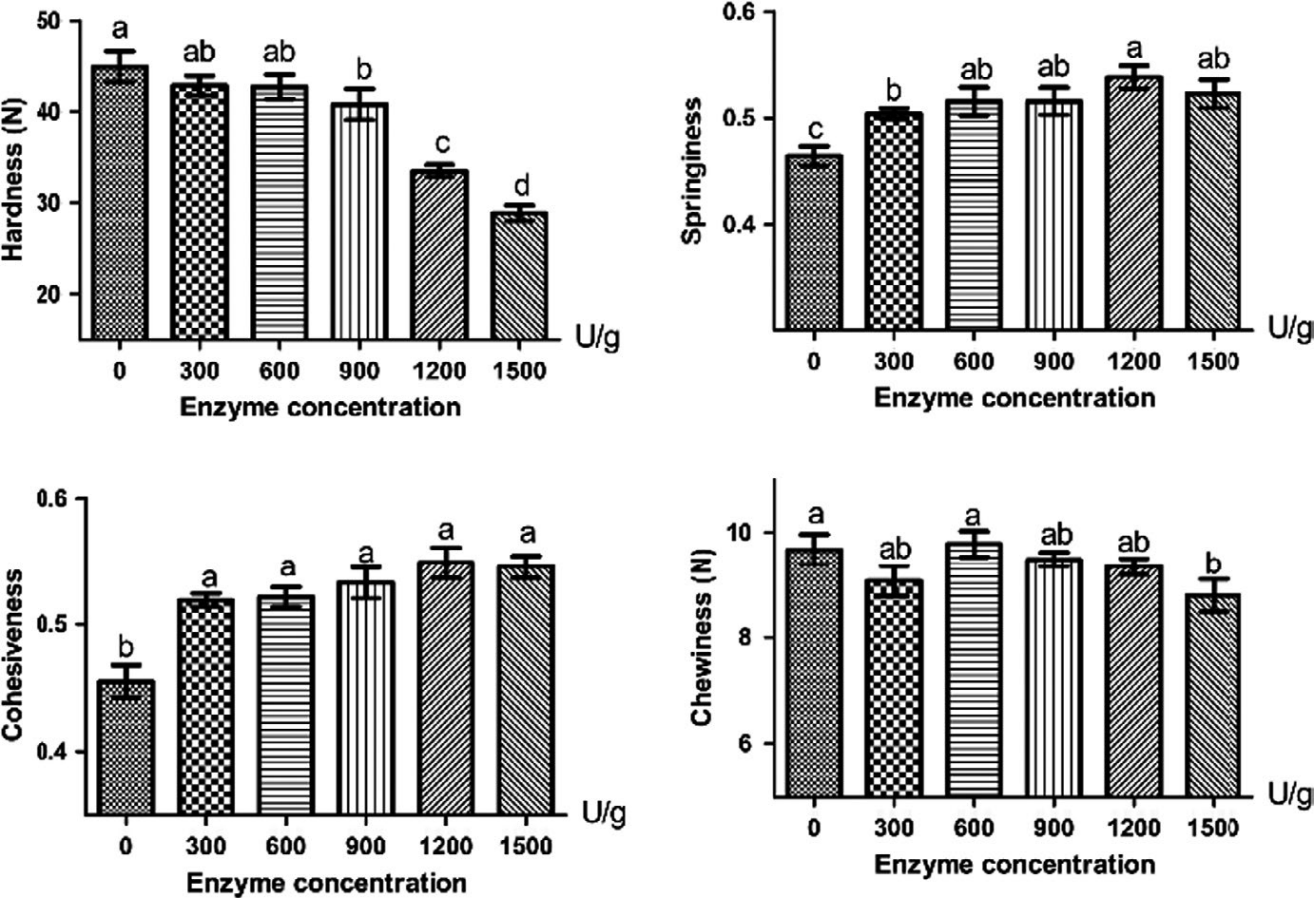
Changes in texture of smoked salted duck. Different superscripts (a, b, c, d) indicate significant differences (*p* < .05)

Compared to the CK, the duck samples treated with 900 U/g (40.81 ± 4.53 N), 1,200 U/g (33.52 ± 1.83 N), and 1,500 U/g (28.88 ± 2.23 N) dose of bromelain showed the significantly decreased hardness values (Figure 3, *p* < .05). The springiness and cohesiveness values were significantly increased in bromelain‐treated groups compared to CK (*p* < .05). As for the chewiness, only the 1,500 U/g treatment group (8.79 ± 0.82 N) was significantly decreased compared to CK (9.67 ± 0.77 N, *p* < .05).

The decreased hardness and increased springiness and cohesiveness values might be due to the effect of bromelain on the damage of myofibrillar structures to generate proteins with low molecular weight (Kemp & Parr, 2012). On the other hands, bromelain degraded duck protein leading to more water binding sites exposed and more interaction between protein and water in the protein–water matrix. It reduced the shear force and hardness and increased the springiness and cohesiveness (Xiong, 2005). The chewiness depended on the strength of the intermolecular bonding of side chains between proteins (Moon, 2018). Therefore, decreasing chewiness in 1,500 U/g group might be due to the excessive hydrolysis of duck protein, which weakened the binding of protein side chains. The similar result was reported in papain‐treated beef samples by Botinestean et al., (2018). As the concentration of papain was 0.3 g/100g meat, the Warner‐Bratzler Shear force was reduced by about 18% compared to the control. Cheng et al., (2020) also found that the treatment of 10 U/g bromelain decreased the shear force of horsemeat from 12.08 kg (the control) to 6.76 kg.

### 3.4 TBARS

The effects of bromelain treatment on the TBARS values of smoked salted duck are shown in Table 2. The TBARS values in 900 U/g (0.40 ± 0.04 mg MDA/kg), 1,200 U/g (0.41 ± 0.06 mg MDA/kg), and 1,500 U/g (0.40 ± 0.02 mg MDA/kg) bromelain–treated samples were significantly lower than CK (0.52 ± 0.09 mg MDA/kg, *p* < .05). Moderate lipid oxidation could improve the flavor, but excessive oxidation would lead to putrid products (Xia et al., 2021). In the current study, the concentration of 900, 1,200, and 1,500 U/g treatments by bromelain in smoked salted duck inhibited the lipid oxidation to a certain extent.

**TABLE 2:** Changes in TBARS of smoked salted duck

Enzyme concentration	0 U/g	300 U/g	600 U/g	900 U/g	1,200 U/g	1,500 U/g
TBARS(mg/kg)	0.51 ± 0.03^a^	0.46 ± 0.01^ab^	0.48 ± 0.02^ab^	0.40 ± 0.01^b^	0.42 ± 0.02^b^	0.40 ± 0.01^b^

Note

Different superscripts (a, b) within a line indicate significant differences (*p* < .05).

Bromelain is an endopeptidase that can extensively hydrolyze protein. Therefore, the decrease of TBARS values might be due to the antioxidant peptides and FAAs produced by the protein degradation from bromelain (Borrajo et al., 2020). It has been reported that peptides and FAAs from bromelain‐induced protein degradation possessed antioxidant activity through reacting with free radicals (Lopez‐Pedrouso et al., 2020). Similar results were also found in sheep muscles when they were treated with ginger protease from ginger extract (Mendiratta et al., 2000). TBARS of sheep muscles treated with 3% ginger protease was reduced to 0.95 mg MDA/kg compared to 1.31 mg MDA/kg in control samples. Feng et al., (2014) also found that treatment with Flavourzyme significantly reduced the TBARS values of Chinese sausage compared to CK.

### 3.5 Electronic tongue detection

The electronic tongue was further used to analyze the taste attribution of different concentrations of bromelain‐treated duck samples (Table 3). As bromelain concentrations increased from 300 to 900 U/g, bitterness values decreased. However, the latter increased as bromelain concentration increased between 1,200 and 1,500 U/g. In addition, the astringency significantly increased in 600, 900, 1,200 and 1,500 U/g groups. However, for saltiness and aftertaste‐B of bromelain marinated ducks, an increase was observed for all treatments in comparison with CK. Likewise, the values of umami of smoked salted duck increased significantly (*p* < .05) by 4%, 6%, 8%, 5%, and 6%, respectively, with increasing bromelain doses. The richness was determined by the effects of bitterness, umami, saltiness, and other parameters. As per the results of this study, it is noteworthy to mention that the highest richness was observed in the 900 U/g group among six treatments.

**TABLE 3:** Changes in electronic tongue detection of smoked salted duck

Enzyme concentration	0 U/g	300 U/g	600 U/g	900 U/g	1,200 U/g	1,500 U/g
Bitterness	4.85 ± 0.11^a^	3.84 ± 0.03^c^	3.81 ± 0.07^c^	3.77 ± 0.17^c^	4.21 ± 0.20^bc^	4.34 ± 0.09^b^
Astringency	0.05 ± 0.01^c^	0.12 ± 0.01^c^	0.27 ± 0.08^bc^	0.67 ± 0.01^b^	1.30 ± 0.11^a^	1.52 ± 0.47^a^
Aftertaste‐B	0.04 ± 0.00^d^	0.49 ± 0.05^bc^	0.27 ± 0.00^c^	0.59 ± 0.07^b^	0.68 ± 0.17^b^	1.00 ± 0.10^a^
Umami	12.79 ± 0.07^c^	13.31 ± 0.15^b^	13.62 ± 0.14^ab^	13.84 ± 0.09^a^	13.46 ± 0.16^b^	13.61 ± 0.04^ab^
Richness	3.58 ± 0.13^b^	3.65 ± 0.12^b^	3.69 ± 0.06^b^	4.53 ± 0.18^a^	3.49 ± 0.15^b^	3.47 ± 0.09^b^
Saltiness	9.04 ± 0.08^c^	9.97 ± 0.16^a^	9.38 ± 0.09^bc^	9.98 ± 0.18^a^	9.82 ± 0.26^ab^	9.78 ± 0.10^ab^

Note

Different superscripts (a, b, c, d) within a line indicate significant differences (*p* < .05).

In this study, the high acceptability in the 900 U/g group compared to CK might be attributed to the sum of FAAs and peptides which could affect the taste. The actions of endopeptidases were found to be distant from carboxyl and amino termini of peptide bonds (Li et al., 2017). These degradation products are mainly water‐soluble nitrogen‐containing compounds that can act as direct or indirect flavor precursors (Zhao et al., 2019). Zhang et al., (2017) found that Flavourzyme promoted the proteolysis of Cantonese bacon as shown by the increasing concentrations of all FAAs detected. Zhao et al., (2020) used bromelain to treat beef muscle and found the types and contents of FAAs were significantly improved, especially glutamicacid, valine, and alanine. Toldra et al., (2020) reported that the type of small peptides was closely related to the presence of flavor. According to other previous studies, it was assumed that the different aromas in meat might be due to differences in the composition of FAAs and peptides (Petrova et al., 2016). For example, leucine and arginine can affect salty taste by interacting with other acids and inorganic salts (Zou et al., 2018). Aspartic acid and serine can increase the umami and sweetness, respectively (Delgado et al., 2020). Methionine can produce low threshold aroma of cooked beef, while isoleucine, phenylalanine, serine, and threonine could produce heterocyclic compounds and Strecker aldehydes, which have unique aroma in meat products (Zhang et al., 2020). Leucine and valine can be the precursors of fragrance and react to produce 2‐methylpropanal and 2‐methylbutanal with cheese flavor and grass flavor. The products of lysine are lactam compounds which have the aroma of barbecue and cooked meat (Keska & Stadnik, 2017). Meanwhile, FAAs and reducing sugars can form aromatic volatiles during cooking (Karpinski et al., 2020).

## 4 CONCLUSIONS

In this study, the marination with bromelain could significantly decrease the values of pH and cooking loss and inhibit lipid oxidation of smoked salted duck. The myosin and the actin were extensively degraded due to bromelain which could damage the integrity of duck meat. Thereby, the hardness was reduced while the springiness and the cohesiveness were significantly increased. At the level of 900 U/g, the richness and the umami of smoked salted duck were enhanced while the bitterness was reduced. In conclusion, the appropriate level of bromelain could improve the eating quality of smoked salted duck, especially at the level of 900 U/g. The effects of bromelain on the composition changes of volatile flavor substances in particular specific FAAs warrant to be further studied.

## CONFLICT OF INTEREST

The authors declare no conflict of interest.

## AUTHOR CONTRIBUTION

**Ziqing Ye:** Conceptualization (equal); Data curation (equal); Formal analysis (equal); Investigation (equal); Methodology (equal); Software (equal); Validation (equal); Writing‐original draft (equal); Writing‐review & editing (equal). **Jian Zhang:** Formal analysis (equal); Methodology (equal); Software (equal). **Jose Manuel Manuel Lorenzo Lorenzo Rodriguez:** Conceptualization (equal); Writing‐review & editing (equal). **Mutian Zhang:** Investigation (equal); Resources (equal). **Wangang Zhang:** Conceptualization (equal); Data curation (equal); Formal analysis (equal); Investigation (equal); Methodology (equal); Resources (equal); Supervision (equal); Validation (equal); Writing‐review & editing (equal).

## ETHICAL REVIEW

This study does not involve any human or animal testing.

## APPENDIX A

**TABLE A1:** The composition of protein of band 1 by NanoLC‐ESI‐MS/MS

Hits	Protein mass	No. of peptide	Sequence header	Protein	Relative abundance	Probability
1	218,369.23	456	>tr|U3J7T0|U3J7T0_ANAPL Uncharacterized protein OS = Anas platyrhynchos OX = 8,839 PE = 3 SV = 1	Uncharacterized protein	20.06%	99.0%
2	206,767.3	433	>tr|U3IWZ4|U3IWZ4_ANAPL Uncharacterized protein OS = Anas platyrhynchos OX = 8,839 PE = 3 SV = 1	Uncharacterized protein	20.01%	99.0%
3	144,246.53	331	>tr|U3ILZ5|U3ILZ5_ANAPL Uncharacterized protein OS = Anas platyrhynchos OX = 8,839 PE = 3 SV = 1	Uncharacterized protein	15.80%	99.0%
4	103,742.01	129	>tr|U3J875|U3J875_ANAPL Uncharacterized protein OS = Anas platyrhynchos OX = 8,839 PE = 3 SV = 1	NA	13.50%	99.0%
5	93,791.66	127	>tr|U3IVM5|U3IVM5_ANAPL Uncharacterized protein OS = Anas platyrhynchos OX = 8,839 PE = 3 SV = 1	NA	11.10%	99.0%
6	225,550.47	116	>tr|U3J280|U3J280_ANAPL Uncharacterized protein OS = Anas platyrhynchos OX = 8,839 PE = 3 SV = 1	Uncharacterized protein	5.40%	99.0%
7	224,490.17	114	>tr|U3IVT5|U3IVT5_ANAPL Uncharacterized protein OS = Anas platyrhynchos OX = 8,839 PE = 3 SV = 1	Uncharacterized protein	6.20%	99.0%
8	166,948.97	84	>tr|U3J2X2|U3J2X2_ANAPL Myomesin 2 OS = Anas platyrhynchos OX = 8,839 GN = MYOM2 PE = 4 SV = 1	Myomesin 2	1.03%	99.0%
9	187,319.22	70	>tr|R0J8F2|R0J8F2_ANAPL Myosin‐6 (Fragment) OS = Anas platyrhynchos OX = 8,839 GN = Anapl_18423 PE = 3 SV = 1	Myosin‐6	4.93%	99.0%
10	183,622.23	15	>tr|U3ITK0|U3ITK0_ANAPL Myomesin 1 OS = Anas platyrhynchos OX = 8,839 GN = MYOM1 PE = 4 SV = 1	Myomesin 1	0.20%	99.0%
11	151,324.13	11	>tr|U3J1L9|U3J1L9_ANAPL Amylo‐alpha‐1, 6‐glucosidase, 4‐alpha‐glucanotransferase OS = Anas platyrhynchos OX = 8,839 GN = AGL PE = 4 SV = 1	4‐alpha‐glucanotransferase	0.13%	99.0%
12	173,606.67	11	>tr|R0JWJ4|R0JWJ4_ANAPL Glycogen debranching enzyme (Fragment) OS = Anas platyrhynchos OX = 8,839 GN = Anapl_05994 PE = 4 SV = 1	4‐alpha‐glucanotransferase	0.14%	99.0%
13	116,408.48	7	>tr|R0LTI7|R0LTI7_ANAPL Sarcoplasmic/endoplasmic reticulum calcium ATPase 2 (Fragment) OS = Anas platyrhynchos OX = 8,839 GN = Anapl_01816 PE = 3 SV = 1	Endoplasmic reticulum class 1/2 Ca(2+) ATPase	0.10%	99.0%
14	129,165.49	5	>tr|U3IHS1|U3IHS1_ANAPL Myosin binding protein C, slow type OS = Anas platyrhynchos OX = 8,839 GN = MYBPC1 PE = 4 SV = 1	Myosin binding protein C1	0.10%	99.0%
15	6,883.76	4	>tr|U3J8G6|U3J8G6_ANAPL Uncharacterized protein OS = Anas platyrhynchos OX = 8,839 PE = 4 SV = 1	Uncharacterized protein	0.30%	99.0%

**TABLE A2:** The composition of protein of band 2 by NanoLC‐ESI‐MS/MS

Hits	Protein mass	No. of peptide	Sequence header	Protein	Relative abundance	Probability
1	40,497.3	137	>tr|U3I8T6|U3I8T6_ANAPL Uncharacterized protein OS = Anas platyrhynchos OX = 8,839 GN = ACTA1 PE = 3 SV = 1	Actin alpha 1, skeletal muscle	58.91%	99.0%
2	42,052.84	99	>tr|A6ZIB9|A6ZIB9_ANAPL Beta‐actin OS = Anas platyrhynchos OX = 8,839 GN = ACTB PE = 2 SV = 1	Beta‐actin	34.93%	99.0%
3	45,065.29	6	>tr|U3ILF5|U3ILF5_ANAPL Phosphoglycerate kinase OS = Anas platyrhynchos OX = 8,839 GN = PGK1 PE = 3 SV = 1	Phosphoglycerate kinase	2.95%	99.0%
4	28,590.79	6	>tr|R4HF03|R4HF03_ANAPL Muscle creatine kinase (Fragment) OS = Anas platyrhynchos OX = 8,839 GN = MCK PE = 2 SV = 1	Creatine kinase	0.80%	99.0%
5	47,763.46	5	>tr|U3IGH1|U3IGH1_ANAPL Creatine kinase, mitochondrial 2 OS = Anas platyrhynchos OX = 8,839 GN = CKMT2 PE = 3 SV = 1	Creatine kinase	0.50%	99.0%
6	49,040.39	4	>tr|U3I342|U3I342_ANAPL Ubiquinol‐cytochrome c reductase core protein 2 OS = Anas platyrhynchos OX = 8,839 GN = UQCRC2 PE = 3 SV = 1	Ubiquinol‐cytochrome c reductase core protein 2	0.30%	99.0%
7	51,592.64	2	>tr|U3I2D1|U3I2D1_ANAPL Ubiquinol‐cytochrome c reductase core protein 1 OS = Anas platyrhynchos OX = 8,839 GN = UQCRC1 PE = 4 SV = 1	Ubiquinol‐cytochrome c reductase core protein 1	0.10%	99.0%
